# Increased annexin A1 and A2 levels in bronchoalveolar lavage fluid are associated with resistance to respiratory disease in beef calves

**DOI:** 10.1186/1297-9716-44-24

**Published:** 2013-04-08

**Authors:** Chandrika Senthilkumaran, Mary Ellen Clark, Khaled Abdelaziz, Ken G Bateman, Allison MacKay, Joanne Hewson, Jeff L Caswell

**Affiliations:** 1 Department of Pathobiology, University of Guelph, Guelph, ON, N1G 2W1, Canada; 2 Pathology Department, Faculty of Veterinary Medicine, Beni-Suef University, Beni-Suef, 62511, Egypt; 3 Department of Population Medicine, University of Guelph, Guelph, ON, N1G 2W1, Canada; 4 Department of Biomedical Sciences, University of Guelph, Guelph, ON, N1G 2W1, Canada; 5 Department of Clinical Studies, University of Guelph, Guelph, ON, N1G 2W1, Canada

## Abstract

Strategies to control bovine respiratory disease depend on accurate classification of disease risk. An objective method to refine the risk classification of beef calves could be economically beneficial, improve welfare by preventing unexpected disease occurrences, refine and reduce the use of antibiotics in beef production, and facilitate alternative methods of disease control. The objective of this study was to identify proteins in bronchoalveolar lavage fluid (BALF) of stressed healthy calves that predict later disease outcome, serve as biomarkers of susceptibility to pneumonia, and play a role in pathogenesis. BALF was collected from 162 healthy beef calves 1–2 days after weaning and transportation. Difference in gel electrophoresis (DIGE) and mass spectrometry were used to compare proteins in samples from 7 calves that later developed respiratory disease compared to 7 calves that remained healthy. Calves that later developed pneumonia had significantly lower levels of annexin A1, annexin A2, peroxiredoxin I, calcyphosin, superoxide dismutase, macrophage capping protein and dihydrodiol dehydrogenase 3. Differences in annexin levels were partially confirmed by western blot analysis. Thus, lower levels of annexins A1 and A2 are potential biomarkers of increased susceptibility to pneumonia in recently weaned and transported feedlot cattle. Since annexins are regulated by glucocorticoids, this finding may reflect individual differences in the stress response that predispose to pneumonia. These findings also have implications in pathogenesis. Annexins A1 and A2 are known to prevent neutrophil influx and fibrin deposition respectively, and may thus act to minimize the harmful effects of the inflammatory response during development of pneumonia.

## Introduction

Shipping fever pneumonia of feedlot cattle results when stress and/or viral infection triggers nasopharyngeal populations of *Mannheimia haemolytica* to proliferate and colonize the lower respiratory tract [1]. The presence of bacteria in the lung elicits an acute inflammatory response characterized by exudation of fluid, formation of fibrin in alveoli, and infiltration of neutrophils and macrophages. These changes lead to diminished lung function, systemic consequences of sepsis, reduced weight gain and feed conversion, reduced carcass quality, and in some cases death.

Contemporary strategies to control losses from shipping fever pneumonia depend on accurate classification of risk groups [2]. Calves that are preconditioned, pre-vaccinated, or from a single source may be at low risk, and appropriate disease control in these calves may be limited to observation of newly arrived cattle and early treatment of clinically ill animals. In contrast, low body weight cattle from multiple sources purchased at auction without reliable health information are at high risk, and metaphylactic antibiotic treatment is often used to control disease in this situation. Imprecise classification of risk groups may result in disease outbreaks in calves wrongly thought to be at low or medium risk, or unnecessary use of antibiotics in calves falsely believed to be at high risk. An objective method to refine the risk classification of feedlot calves could be economically beneficial, improve welfare by preventing unexpected disease occurrences, refine and reduce the use of antibiotics in beef production, and facilitate alternative methods of disease control.

Bronchoalveolar lavage fluid contains cellular and soluble material from the distal bronchi, bronchioles, and alveoli, and provides an opportunity to sequentially study host factors and pathogens during development of naturally occurring disease [3-5]. The objective of the present study was to identify proteins in BALF samples obtained from clinically healthy calves soon after arrival to a feedlot, and correlate differences in these proteins with later development of pneumonia. Identifying such proteins would be useful as diagnostic biomarkers of disease susceptibility: although it is probably not practical to routinely collect BALF as a diagnostic sample in commercial feedlots, it is expected that protein changes in BALF may follow similar patterns in more easily obtained diagnostic samples. Further, comparing differences in BALF proteins should reveal novel factors that influence which calves in a population develop disease and which remain healthy, thereby providing a new window on understanding the complex pathogenesis of this disease.

## Materials and methods

### Study design and sample collection

Animal use was approved by the Animal Care Committee of the University of Guelph, 07R086. A total of 162 male beef calves, in five different groups from different sources in Ontario, were weaned in October and November, transported soon after weaning for a duration of 1.5 to 6 h, and housed at the Elora Beef Research Station, University of Guelph. Within 2–3 days of arrival, the rectal temperature, weight and overall body condition were recorded, and samples of BALF and jugular venous blood were collected from each calf [4]. Blood samples were analyzed by the Animal Health Laboratory, University of Guelph, for serum haptoglobin [4], serum cortisol (competitive chemiluminescent enzyme immunoassay, Immulite 1000 Cortisol, Siemens Healthcare Diagnostics) and complete blood counts (citrate-anticoagulated whole blood). This time point is referred to as the on-arrival period. At this time, some calves had nasal discharge, and bronchoscopy revealed hyperemia of the upper respiratory tract mucosa, but there was no fever or clinical signs of pneumonia (as defined below).

Calves were monitored for the next six to seven weeks for the development of clinical signs. Each calf with pneumonia, as well as 2–3 clinically healthy calves from the same pen, were examined clinically and serum samples were collected for later measurement of haptoglobin. BALF samples were not collected at this time. Pneumonia was detected by the experienced facility operator and confirmed by a veterinarian. Inclusion criteria were: (1) clinical signs such as depression, drooping of the head or ears, failure to approach the food bunk, lack of rumen fill, or standing apart from the group, (2) no evidence of an alternative disease other than pneumonia, (3) rectal temperature ≥ 40.2°C and (4) serum haptoglobin level ≥ 2.0 g/L. Retrospectively, 12 calves met these inclusion criteria as “Calves that later developed pneumonia”. Calves used for DIGE analysis (see below) developed pneumonia at 5, 5, 14, 15, 15, 50, and 62 days after initial sampling.

For each calf diagnosed with pneumonia, two to three healthy calves from the same pen were selected as possible controls. Inclusion criteria for the healthy group were: (1) matching to the same pen as a case of pneumonia, (2) lack of clinical abnormalities including evidence of respiratory disease, (3) rectal temperature ≤ 40°C, and (4) serum haptoglobin levels < 1.0 g/L (all but one were < 0.5 g/L). Calves selected as controls were monitored throughout the study period; those that later developed illness were excluded. Retrospectively, 10 calves met these inclusion criteria as “Calves that remained healthy”. Seven animals in each group were randomly selected for the subsequent analysis.

### DIGE analysis of BALF

BALF collected during the on-arrival period was transported to the laboratory on ice within 1–2 h of sampling, filtered through gauze, then centrifuged at 5000 × *g* for 45 min at 4°C, and the supernatants were stored at −80°C for later use. After the clinical observation period of 6–7 weeks, BALF samples were processed from those calves that were assigned to the study groups “Calves that remained healthy” and “Calves that later developed pneumonia”. BALF samples were concentrated by centrifugal filtration using 5 kDa nominal molecular weight limit devices (Amicon® Ultra-15, Millipore, Bedford, MA, USA). Interfering substances in the samples were removed using ReadyPrep™ 2-D Cleanup Kit (Bio-Rad Laboratories, Hercules, CA, USA). The cleaned protein pellets were solubilized in 25 to 50 μL of lysis buffer (8 M urea, 4% CHAPS, 30 mM Tris-Cl, pH 8.5), total protein was quantified by a colorimetric assay (2D Quant Kit, GE Healthcare Bio-Sciences, Piscataway, NJ, USA), and the sample was stored at −20°C for subsequent labeling.

The protein content of BALF preparations was evaluated by DIGE using 7 analytical gels. The total protein loaded onto each analytical gel included 25 μg of protein from the BALF of a calf that remained healthy, 25 μg protein from a calf that later developed pneumonia, and 25 μg of a pooled sample from all 14 animals that served as the internal control. These protein samples were labeled with 100 pmol each of Cy3, Cy5, or Cy2 dyes respectively, following the manufacturer’s protocol (GE Healthcare, Montreal, QC, Canada). The 7 preparations, each containing 75 μg of protein, were mixed with rehydration buffer (8 M urea, 1% CHAPS, 65 mM dithiothreitol (DTT), and 0.5% v/v immobilized pH gradient (IPG) buffer pH 3–10). Isoelectric focusing was carried out on 13 cm, non-linear pH 3–10 Immobiline DryStrips (GE Healthcare, Montreal, QC, Canada) at 20°C on an Ettan IPGphor Isoelectric Focusing System (GE Healthcare, Montreal, QC, Canada) as follows: strip rehydration for 10 h, then 300 V for 30 min, 800 V for 30 min, 2000 V for 5 h, 3500 V for 2 h, and 5000 V for 14 h until 50 000 volt-hours were achieved at 50 μA/strip.

After isoelectric focusing, strips were either stored at −20°C, or prepared directly for the second dimensional separation by equilibrating for 15 min in a 5 mL equilibration buffer containing 6 M urea, 30% glycerol, 2% sodium dodecyl sulfate (SDS), 75 mM Tris-hydrochloride (pH 8.8), 0.005% bromophenol blue and freshly added 1% (w/v) DTT; and subsequently for 15 min in another 5 mL equilibration buffer which had freshly added 2.5% (w/v) iodoacetamide (Sigma-Aldrich Canada Ltd., Oakville, ON, Canada). For the 2nd dimension, pH gradient strips were applied directly onto individual 12% SDS-polyacrylamide, non-gradient, 20 × 25 cm gels on a DALT6 electrophoresis unit (GE Healthcare, Montreal, QC, Canada), at 3 W/gel overnight and changed to 6 W/gel in the morning until the bromophenol blue dye front was 2 cm from the gel end. Kaleidoscope 10–250 kDa molecular weight markers (Bio-Rad Laboratories, Hercules, CA, USA) were used as reference. Gels were scanned with a Typhoon 9410 variable mode imager (GE Healthcare, Montreal, QC, Canada) by excitation/emission wavelengths specific for Cy2 (488 nm/520 nm), Cy3 (532 nm/580 nm), and Cy5 (633 nm/670 nm).

Statistical and quantitative analyses of protein spot concentrations on multiplexed DIGE images were completed by DeCyder 6.5 software (GE Healthcare, Montreal, QC, Canada). Measurements of spot abundance were normalized to the pooled internal control, and the ratio of protein abundances in the two sample groups was calculated for samples on each analytical gel (intra-gel analysis) by differential in-gel analysis. The biological variation analysis module was used for inter-gel matching of internal standard and samples across all gels, and for the comparative cross-gel statistical analyses of all spots. All protein spots detected were examined manually and artifacts were removed. Differentially expressed protein spots were defined as those for which the differences in the normalized spot volume between the two groups (calves that remained healthy compared to those that later developed pneumonia) was ≥ 2-fold and significantly different at *P* < 0.05.

In order to retrieve the selected spots for mass spectrometry, three of the seven gels were randomly selected, post-stained with Sypro Ruby dye (Bio-Rad Laboratories, Hercules, CA, USA), and scanned with a Typhoon 9410 instrument at 532 nm with 610 nm filters. Protein spots of interest were excised from the gel automatically using an Ettan Spot Picker robot (GE Healthcare, Montreal, QC, Canada). Those spots were digested and analyzed by tandem mass spectrometry at the Mass Spectrometry Facility, Hospital for Sick Children, Toronto, ON, Canada [6], and proteins were identified (Scaffold Proteome Software, version 02, Portland, OR, USA). All MS/MS spectra were searched against the bovine protein database (downloaded 11 March 2011 from the National Center for Biotechnology Information), and peptides were identified based on the coverage, probability, peptide match, amino acid match, similarity of the observed and theoretical molecular weights as well as the *pI* and the unique total spectra.

### Western blot

Three calves from each group used in the DIGE experiment were arbitrarily selected for the Western blot study. BALF was cleaned and concentrated as previously described. Samples containing 60 μg of total protein for annexin A1, or 40 μg of total protein for annexin A2, were incubated for 5 min at 95°C in SDS loading buffer containing 65 mM DTT, then separated on a 12% SDS gel using 100 V for 90 min. The gel was transferred to a polyvinyl difluoride transfer membrane (Immobilon-P^SQ^, Millipore Corporation, Billerica, MA) for 90 min at 90 V in transfer buffer containing 0.192 M glycine and 0.025 M Tris in 20% methanol. The membrane was placed on a filter paper, dried 1 h at 37°C, rewetted in 100% methanol for 5 min, washed with Tris PBS and 0.1% Tween for 15 min, and blocked overnight with 5% BSA at 4°C. Lysates of HEK293T cells expressing human annexins AI and A2 (NBL1-07557 and NBL1-07561, Novus Biologicals, Oakville, ON, Canada) were used as positive controls, and the corresponding empty plasmid vectors were used as negative controls.

For detection of annexin A1, the membrane was incubated for 1 h with goat anti-human annexin A1 antibody (1:200; NBP1-50411, Novus Biologicals, Oakville, ON, Canada), then for 30 min with peroxidase-conjugated rabbit anti-goat secondary antibody (1:2000; DakoCytomation, Glostrup, Denmark), and bands were detected by chemiluminescence (ECL Plus, GE Healthcare) visualized with a ChemiDoc system (BioRad, Mississauga, Ontario, Canada). For detection of annexin A2, membranes were incubated for 1 h with rabbit anti-human annexin A2 antibody (1:100; NBP1-59124, Novus Biologicals, Oakville, ON, Canada), then for 30 min with peroxidase-conjugated goat-anti rabbit secondary antibody (1:2000; DakoCytomation, Glostrup, Denmark), and bands were detected as above.

### Statistical analysis

The data were collected in the form of an incomplete block design, where the block was the truckload of animals, with sub-sampling of the two outcome groups. Data were analyzed using Proc Mixed (SAS 9.2, SAS Institute Inc., Cary, NC). To assess the ANOVA assumptions, comprehensive residual analyses were performed. The assumption of normality was tested using the four tests offered by SAS (Shapiro-Wilk, Kolmogorov-Smirnov, Cramervon Mises, and Anderson-Darling tests). Blood neutrophil counts were not normally distributed, and the data were log transformed. In addition, residuals were plotted against the predicted values and explanatory variables used in the model to reveal any outliers, bimodal distributions, or the need for data transformations. Data are expressed as means ± standard error of mean (SEM). Differences were considered significant at *P* ≤ 0.05.

## Results

### Clinical and hematologic findings

Temperature, body weight and the complete blood counts were measured 2–3 days after arrival, at the time of bronchoalveolar lavage. There were no significant differences in temperature or body weight between the two groups (*P* = 0.97 and 0.60, respectively), in this on-arrival period. Although the numbers of total leukocytes, neutrophils, lymphocytes and monocytes were lower in calves that later developed pneumonia compared to calves that remained healthy, these differences were not statistically significant (*P* = 0.36, 0.78, 0.40 and 0.91, respectively).

### DIGE analysis of BALF

**Figure 1:**
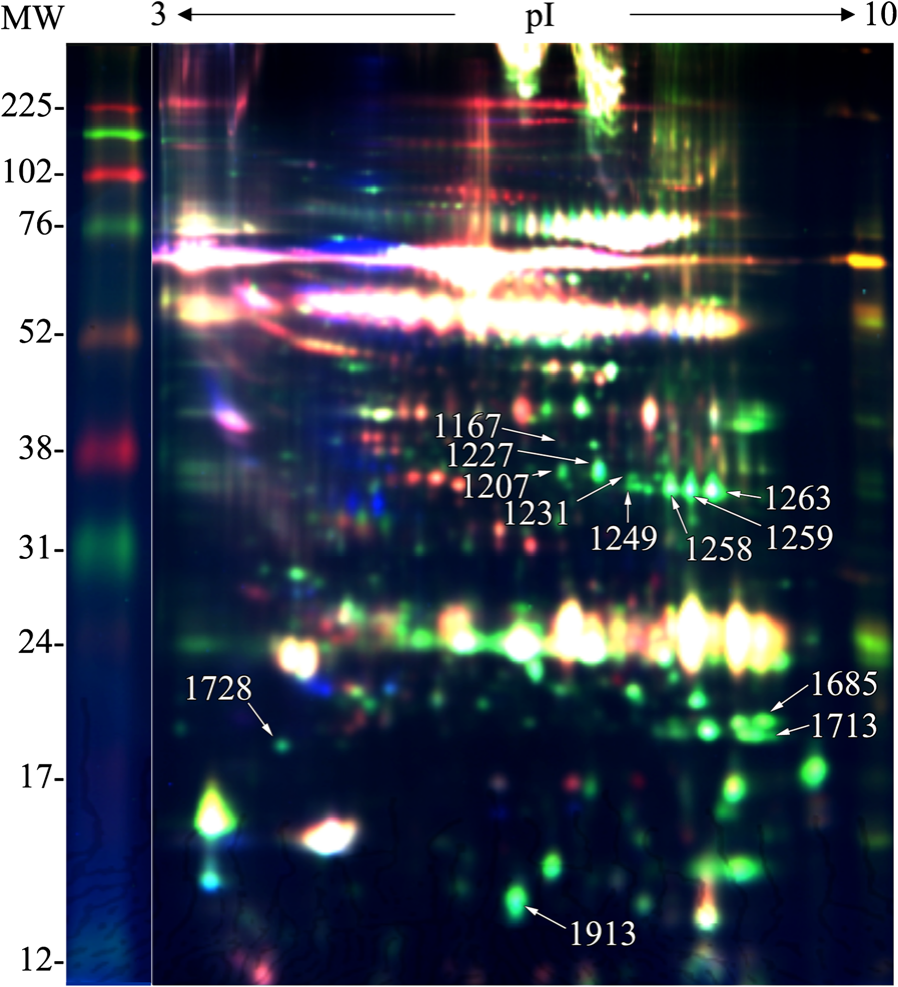
**A representative overlay of three dye scans of the DIGE analysis of pooled bovine BALF.** Protein spots that were more abundant in calves that remained healthy versus those that later developed pneumonia are labeled. 1167: macrophage capping protein; 1207, 1227 and 1231, isoforms of annexin A1; 1249 and 1258, isoforms of annexin A2; 1259 and 1263, isoforms of dihydrodiol dehydrogenase 3; 1685 and 1713, isoforms of peroxiredoxin I; 1728, calcyphosin; 1913, superoxide dismutase chain B.

The DIGE gels of BALF from clinically healthy calves, obtained within 2–3 days of arrival to the feedlot, contained approximately 2400 identifiable protein spots that ranged in molecular weight from 12–225 kDa across the pI range of 3–10 (Figure 1). Among these, the normalized spot volumes of 27 were significantly greater (*P* < 0.05) and ≥ 2-fold higher in calves that remained healthy compared to those calves that later developed pneumonia. The normalized volume of some spots were higher in calves that later developed pneumonia compared to those that remained healthy, but this difference was never ≥ 2-fold and the *P* value was greater than 0.05 for some, so these spots were not studied further. Of the 27 differentially expressed spots, 15 were too faint to be reliably identified in Sypro Ruby-stained gels. The other 12 spots were analyzed by tandem mass spectrometry (Table 1). Five of those 12 spots proved to be annexins A1 or A2.

**Table 1:** Mass Spectrometry data for protein spots from DIGE analysis of BALF sampled within 2–3 days of arrival to the feedlot, for which spot densities were greater in calves that remained healthy compared to those that later developed pneumonia

**Spot #**	**Protein identified**	**Acc #**^**a**^	**Cov %**^**b**^	**Prob**^**c**^	**Pep match**^**d**^	**AA**^**e**^	**MW**^**f **^**(kDa)**	**PI**^**g**^	**Fold increase**	***P *value**
1167	Macrophage capping protein	gi30466254	20	100	6	71/349	40/39	6/5.3	2.01	0.020
1207	Annexin A1	gi74	62	100	21	213/346	38/39	6.0/6.4	1.94	0.027
1227	Annexin A1	gi74	55	100	18	190/346	38/39	6.2/6.4	2.02	0.030
1231	Annexin A2	gi27807289	23	100	8	77/339	37/39	6.4/6.9	2.33	0.036
1249	Annexin A2	gi27807289	42	100	13	143/339	37/39	6.2/6.9	1.94	0.007
1258	Annexin A2	gi27807289	51	100	17	172/339	37/39	6.6/6.9	2.24	0.018
1259	Dihydrodiol dehydrogenase 3	gi3079344	30	100	12	98/323	37/37	6.8/7.6	2.69	0.018
1263	Dihydrodiol dehydrogenase 3	gi3079344	41	100	11	132/323	37/37	7/7.6	2.91	0.022
1685	Peroxiredoxin I	gi66773956	41	100	9	82/199	20/22	7.3/8.6	2.69	0.004
1713	Peroxiredoxin I	gi66773956	45	100	10	90/199	20/22	7.4/8.6	2.31	0.038
1728	Calcyphosin	gi115497428	38	100	7	72/189	21/21	4.8/4.8	2.62	0.008
1913	Superoxide dismutase, chain B	gi12084767	23	100	3	35/152	15/16	5.8/5.7	1.89	0.022

^a^ Acc #: Accession number. The number associated with a protein in a FASTA database, that Scaffold software uses to link to information about that protein on the NCBI website.

^b^ Cov %: Percent coverage; percentage of the predicted peptide identified by the mass spectrometry data.

^c^ Prob: The calculated probability of that a given protein has been identified correctly.

^d^ Pep match: Number of unique peptides on which the protein identification was based.

^e^ AA: Number of amino acids identified / total number of amino acids in the protein.

^f^ MW: Observed / theoretical molecular weight of the protein in kilodaltons.

^g^ pI: Observed / theoretical isoelectric point of the protein.

Cortisol concentrations were measured in serum samples collected at the on-arrival time point. Serum cortisol concentrations were correlated with annexin A1 levels in BAL fluid (Pearson r = 0.56, *P* = 0.038, N = 14). Serum cortisol concentrations were higher in calves that remained healthy (140.8 ± 16.01 nmol/L, N = 10) compared to those that later developed pneumonia (111.0 ± 10.97 nmol/L, N = 12), although this difference was not significant (*P* = 0.1309, Student’s *t*-test).

### Western blot

**Figure 2:**
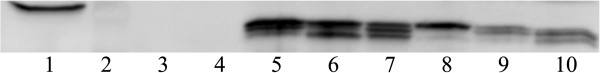
**Western blot analysis of BALF samples labeled with antibody to annexin A1.** Lane 1: synthetic annexin A1 peptide (positive control), 2: molecular weight ladder, 3: blank, 4: negative control, 5–7: samples from calves that remained healthy, 8–10: samples from calves that later developed pneumonia. The three bands indicate that there are three isoforms of the annexin A1 present in BALF, and the density of the bands is higher in the calves that remained healthy compared to the calves that later developed pneumonia.

**Figure 3:**
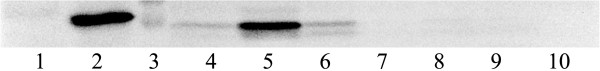
**Western blot of BALF samples labeled with antibody to annexin A2.** Lane 1: synthetic annexin A1 peptide, 2: synthetic annexin A2 peptide (positive control), 3: molecular weight ladder, 4–6: samples from calves that remained healthy; 7–9: samples from calves that later developed pneumonia, 10: negative control. The two bands indicate that there are two isoforms of the annexin A2 present in BALF, and the density of the bands is higher in the calves that remained healthy compared to the calves that later developed pneumonia.

Western blot analysis of annexin A1 and annexin A2 were used to validate the DIGE analysis (Figures 2 and 3). The blot probed with antibody to annexin A1 revealed three distinct bands, with molecular weights of approximately 37 kDa (Figure 2). These three bands were generally more dense in samples from the calves that remained healthy compared to those from calves that later developed pneumonia, but there was variability in the number and density of individual bands between animals (Figure 2). The blot probed with antibody to annexin A2 had two distinct bands with molecular weights of approximately 37 kDa. Of the six calves tested by western blot, these two bands were present in all three calves that remained healthy, but absent in all three calves that later developed pneumonia. The density of the annexin A2 bands were variable, in the calves that remained healthy (Figure 3). Annexin A1 used as a positive control in the same blot was not labeled by the antibody to annexin A2.

Based on the mass spectrometry analysis of the DIGE data, three spots were identified as annexin A2 and two spots were identified as annexin A1 (Table 1). Spot #1231 was first identified as annexin A2 based on the mass spectrometry data (Table 2), but the western blot showed three bands for annexin A1 and two bands for annexin A2 (Figures 2 and 3). Thus, it is likely that spot #1231 either represents an isoform of annexin A1, or superimposed isoforms of both annexin A1 and A2.

**Table 2:** Mass spectrometry data for protein spot 1231

**Spot #**	**Protein identified**	**NCBI**^**a **^**Acc. NO**	**Cov**^**b **^**%**	**Pro**^**c**^	**Pep**^**d **^**match**	**AA**	**MW**^**e **^**(KDa)**	**PI**^**f**^	**Unique/**^**g **^**Total spectra**
1231	Annexin A1	gi74	16	100	5	56/346	38/39	6.4/6.4	5/5
1231	Annexin A2	gi27807289	23	100	8	77/339	37/39	6.4/6.9	8/8

^a-f^ Abbreviations are the same as for Table 1.

^g^ Number of unique spectra identified for the spot / total spectra identified for the spot.

## Discussion

The goal of this study was to identify a biomarker that predicts susceptibility to bovine respiratory disease in recently weaned and transported calves, at the time they arrive at the feedlot. Since the ideal biomarker would also play a mechanistic role in conferring susceptibility or resistance to disease, a second goal was to identify novel proteins in the epithelial lining fluid of the lower respiratory tract that determine susceptibility to pneumonia. We identified several proteins that were present at higher levels in the calves that remained healthy, compared to the calves that later developed pneumonia, and most of these proteins are known to function in resolution of inflammation. Among these proteins, annexin A1 and A2 showed a strong association with disease outcome, are known to be regulated by stress and glucocorticoids, may have a unique role in the pathogenesis of bovine respiratory disease, and are potential biomarkers for susceptibility to this disease in recently arrived, stressed feedlot cattle.

The study population was representative of feedlot production practices in much of North America, as the calves had been abruptly weaned in October and November, and transported to a different facility from five different sources. However, it should be acknowledged that the transportation time was 6 h or less, calves were not purchased through an auction, and mixing of calves from different sources was limited. Antimicrobial treatment was not given until clinical signs developed, so the findings are most relevant to disease control strategies that avoid metaphylactic use of antimicrobial drugs.

Calves were considered to be clinically healthy at the time of sampling, 2–3 days after arrival. Although nasal discharge and hyperemia of the nasal and tracheal mucosa was observed in a few animals, clinical signs of pneumonia were not detected at this on-arrival time point, and none of these calves had elevations in rectal temperature, serum haptoglobin concentrations, or blood neutrophil counts.

Clinical diagnosis of pneumonia is problematic in cattle [7]. In this study, the positive and negative selection criteria were based on evaluation of clinical signs by the experienced feedlot operator, confirmation of the clinical diagnosis by a veterinarian, elevated rectal temperature, and increased serum haptoglobin levels. Calves were only included in the study groups “Later developed pneumonia” or “Calves that remained healthy” if all of these factors were present or absent, respectively, to increase confidence in the diagnosis. Other calves had conflicting clinical and laboratory data, and were not included in the DIGE analysis.

Although we recognize that the difficulty in acquiring BALF makes it unsuitable for routine screening of feedlot cattle, our analysis was based on BALF because it is the more appropriate sample for detecting changes in the epithelial lining fluid of the distal respiratory tract [4,8,9]. Initial work was done with 2-dimensional electrophoresis (data not shown), but DIGE was used in the present study because of superior sensitivity and reproducibility for the identification of proteins in the BALF.

Among those differentially expressed proteins, annexin A1 and A2 were of particular interest. Other differentially expressed proteins were macrophage capping protein, calcyphosin, dihydrodiol dehydrogenase 3, peroxiredoxin I, and superoxide dismutase. Three of these are antioxidants: dihydrodiol dehydrogenase 3 is an aldoketoreductase that maintains cellular redox homeostasis, peroxiredoxin I catalyzes hydrogen peroxide and also has anti-inflammatory functions, and superoxide dismutase scavenges superoxide anion and attenuates lung inflammatory responses [10]. Since the lung encounters considerable oxidative stress, animals having higher levels of these antioxidants and anti-inflammatory proteins may be protected from tissue damage caused by bacterial infection. Macrophage capping protein, also named Cap G and cofilin, is a calcium-sensitive protein expressed in alveolar macrophages [11,12], and has roles in rearrangement of actin filaments, membrane ruffling, and complement- and immunoglobulin-mediated phagocytosis [13]. Calcyphosin is a calcium-binding protein of uncertain function: it inhibits mast cell degranulation, has anti-allergic properties in bovine lung [14], and is involved in cAMP signaling [15], cell proliferation and differentiation [16].

The differential expression of annexins A1 and A2 were of particular interest, because of the number of differentially expressed spots corresponding to these proteins, the magnitude and consistency of the differences, and the apparent relevance to the pathogenesis of bovine respiratory disease. Annexins A1 and A2 were found at higher levels in the BALF of calves that remained healthy compared to those that later developed pneumonia, and these DIGE and mass spectrometry findings were confirmed by Western blot analysis of the same BALF samples. The Western blot analysis revealed 3 isoforms of annexin A1 and two isoforms of annexin A2. This preliminary analysis also revealed considerable variation between animals in annexin A1 and A2 isoforms, and additional investigations will be needed to determine the reasons for this variability and the relationship with disease susceptibility.

Annexins A1 and A2 are expressed in bronchial epithelial cells of cattle [17]. Annexins are also found in extracellular tissue fluids, and annexin A2 is detected on the surface of endothelial cells [18,19]. Glucocorticoids induce expression of both the annexin A1 gene and the annexin receptor ALXR [20,21], and effect rapid translocation of annexin A1 to the cell surface [22]. Similarly, annexin levels are greater in hyperadrenocorticism and in response to stress [23,24]. Annexin A1 mediates some of the anti-inflammatory effects of glucocorticoids: it binds to the surface of neutrophils, inducing loss of L-selectin and neutrophil apoptosis [25], and acts on macrophages to promote phagocytosis and removal of apoptotic cells [26]. These functions of annexin A1 reduce neutrophil extravasation and accumulation in the tissue [23].

Of relevance to feedlot cattle, we previously found that annexin A1 increased significantly in the BALF of beef calves after transportation stress [4], and a preliminary analysis in the current study confirmed a positive correlation between serum cortisol concentration and annexin A1 levels in BALF. Thus, it is plausible that annexin A1 may be a biomarker of stress-associated susceptibility to bovine respiratory disease. However, we found increased levels of annexin A1 in calves that remained healthy compared to those that later developed disease, yet glucocorticoids are expected to induce annexin expression. Therefore, stress does not simply result in both annexin A1 induction and increased susceptibility to pneumonia; there is likely a more complex relationship between stressful experiences, synthesis of glucocorticoids, levels of annexin A1 in the epithelial lining fluid of the airways, and susceptibility to pneumonia.

Annexin A2 plays a vital role in several biological processes, including matrix degradation [27], preventing angiogenesis [28], cellular protection against oxidative injury [29], repair of airway epithelial cells [30], and the pro-inflammatory effects of tissue plasminogen activator on macrophages [31]. However, the most recognized role of annexin A2 is in promoting fibrinolysis. Annexin A2 is a receptor for plasminogen and tissue plasminogen activator on the surface of endothelial cells, resulting in activation of plasmin and initiation of fibrinolysis [32].

These findings of higher levels of annexins A1 and A2 in calves that remained healthy after arrival to the feedlot suggest a role of annexins in the pathogenesis of bovine respiratory disease. During *M. haemolytica* pneumonia, there is a strong influx of neutrophils and macrophages, and accumulation of fibrin in the lung. These cascades of events lead to neutrophil-induced tissue injury, and impair alveolar ventilation and gas exchange [1]. The data suggest that calves with high levels of annexin A1 may be better able to control neutrophil infiltration and promote neutrophil apoptosis in the lung, which could prevent neutrophil-induced lung injury and dampen the systemic consequences of endotoxemia. Similarly, the findings suggest that calves having higher levels of annexin A2 may be better able to degrade the fibrin that forms within the pulmonary alveoli, which may reduce the severity or progression of disease following *M. haemolytica* infection.

The present study was not designed to identify the mechanism for differing annexin levels in susceptible vs. resistant calves. Additional work will be needed to evaluate the cellular source of annexins in the lung, the effect of genetics and individual differences on baseline levels of annexin proteins, and the impact of infection and weaning/transportation stress on annexin levels. Thus, characterizing what role these differing annexin levels have in the pathogenesis of bacterial pneumonia will require additional investigation. Nonetheless, annexins A1 and A2 show promise as novel biomarkers of disease susceptibility in recently arrived feedlot cattle. Such biomarkers could be useful for quantification of stress responses under varying management conditions, and accurate classification of recently arrived feedlot cattle with respect to risk of bovine respiratory disease, to facilitate group-specific disease control strategies.

## Abbreviations

BALF: Bronchoalveolar lavage fluid; DIGE: Difference in gel electrophoresis; DTT: Dithiothreitol; SDS: Sodium dodecyl sulfate; SEM: Standard error of mean.

## Competing interests

The authors declare that they have no competing interests.

## Authors’ contributions

CS carried out the studies and prepared the manuscript. MEC contributed to development of methods, and acquisition and analysis of the data. KA and JH contributed to study design and acquisition of clinical samples. KGB participated in study design. JLC conceived of the study and participated in its design and coordination and helped to draft the manuscript. All authors read and approved the final manuscript.
